# Sitting supracerebellar infratentorial approach for resection of posterior fossa arteriovenous malformation

**DOI:** 10.3171/2020.10.FOCVID2091

**Published:** 2021-01-01

**Authors:** Evan Joyce, Ramesh Grandhi, William T. Couldwell

**Affiliations:** Department of Neurosurgery, Clinical Neuroscience Center, University of Utah, Salt Lake City, Utah

**Keywords:** arteriovenous malformation, supracerebellar infratentorial approach, posterior fossa

## Abstract

Arteriovenous malformations (AVMs) of the posterior fossa represent just 5%–15% of all intracranial AVMs. Rupture often leads to devastating brainstem compression, with mortality reported as high as 67%. A life-saving decompressive craniectomy with or without hematoma evacuation may be necessary in the acute setting to alleviate mass effect before proceeding with definitive treatment of the vascular pathology. Here, the authors demonstrate the utility of using a generously sized temporizing decompressive suboccipital craniectomy to subsequently allow for a more judicious resection of a Spetzler-Martin grade III AVM fed by the right superior cerebellar artery using a sitting supracerebellar infratentorial approach.

The video can be found here: https://youtu.be/L195wmw3p_4

## Transcript

**0:28** This is a case of a 63-year-old woman who had a devastating intraparenchymal hemorrhage within the cerebellum from a Spetzler-Martin grade III arteriovenous malformation.

**0:41** This is the presenting CT. She was taken emergently for a life-saving decompression, but the CTA had demonstrated a known AVM, just behind the brainstem at the mesencephalic-pontine junction. It was fed predominantly from the superior cerebellar artery on the right side, with the draining vein on the dorsum of the cerebellum going to the region of the torcula.

**1:07** We took her to the operating room, and 2.5 weeks later, do a sitting position, and use the previous decompression incision to open up and expose the region of the posterior fossa. We’ll extend the craniectomy above the region of the torcula to allow us an unimpeded view on the dorsum of the cerebellum.

**1:30** The dura is opened and elevated. A midline draining vein is identified and divided, but this is not the draining vein for the AVM. We’re going predominantly on the left side, as the draining vein from the AVM is on the right side of the dorsum of the cerebellum.

**1:51** We open up the roof of the cerebellum and identify the clot. After decompression of the hematoma, we identify the small AVM on its posterior aspect and dissect along the left side of the AVM, finding the interface between the cerebellum parenchyma and the AVM directly. The vessels are isolated, cauterized, and divided.

**2:22** We continue our dissection anteriorly and find the superior cerebellar artery on the patient’s left side, and carefully dissect and identify the feeding small arteries to the AVM from the native superior cerebellar artery. Now we’re coming along the anterior aspect of the AVM adjacent to the brainstem, taking care to divide all the feeding vessels but not embarrass the superior cerebellar arteries. The superior cerebellar arteries are left intact. This is the roof and the region of the venous confluence. We continue dissecting the AVM off of the back of the region of the brainstem, again carefully preserving the superior cerebellar artery branches.

**3:26** Now we’ll come along the right side of the AVM and dissect it from the cerebellar parenchyma on the right. We continue our dissection anteriorly. You can see the right superior cerebellar artery, and we’ll identify the draining vein as the final step in removal of the AVM. The AVM draining vein is cauterized and divided. The lesion is removed. We obtain hemostasis and inspect and ensure that there is no residual AVM along the posterior aspect of the brainstem.

**4:10** The hematoma cavity is covered with Surgicel, and the dura is closed with a dural substitute. The muscle is then closed in separate layers, and the skin is closed with a running nylon suture. Postoperatively, she improved significantly, and her postoperative CT shows complete resection of the hematoma, and the angiogram demonstrates no further AVM visualized. There is no early draining veins. She continued to make a good neurological recovery.^1–5^

## Author Contributions

Primary surgeon: Couldwell, Grandhi. Editing and drafting the video and abstract: Couldwell, Joyce. Critically revising the work: Couldwell, Joyce. Reviewed submitted version of the work: Couldwell, Joyce. Approved the final version of the work on behalf of all authors: Couldwell. Supervision: Couldwell.
